# Blood donation practices and behavioral intentions: A scoping review using the theory of planned behavior

**DOI:** 10.1371/journal.pone.0333426

**Published:** 2026-03-12

**Authors:** Gebeyehu Lakew, Bisrat Tewelde Gebretsadkan, Gebrie Getu Alemu, Astewil Moges Bazezew, Amlaku Nigusie Yirsaw, Wubet Tazeb Wondie, Berihun Agegn Mengistie, Tenagnework Eseyneh Dagnaw, Mekuriaw Nibret Aweke, Nebebe Demis Baykemagn

**Affiliations:** 1 Department of Health Promotion and Health Behavior, Institute of Public Health, College of Medicine and Health Sciences, University of Gondar, Gondar, Ethiopia; 2 Department of Environmental Health and Behavioral Sciences, School of Public Health, College of Medicine and Health Sciences, Mekelle University, Mekelle, Ethiopia; 3 Department of Epidemiology and Biostatistics, Institute of Public Health, College of Medicine and Health Sciences, University of Gondar, Gondar, Ethiopia; 4 Department of Surgical Nursing, School of Nursing, College of Medicine and Health Sciences, University of Gondar, Gondar, Ethiopia; 5 Department of Pediatrics and Child Health Nursing, College of Health Sciences and referral Hospital, Ambo University, Ambo, Ethiopia; 6 Department of General Midwifery, School of Midwifery, College of Medicine and Health Sciences, University of Gondar, Gondar, Ethiopia; 7 Department of Public Health, College of Medicine and Health Sciences, Injibara University, Injibara, Ethiopia; 8 Department of Human Nutrition, Institute of Public Health, College of Medicine and Health Sciences, University of Gondar, Gondar, Ethiopia; 9 Department of Health Informatics, Institute of Public Health, College of Medicine and Health Sciences, University of Gondar, Gondar, Ethiopia; Universiti Malaysia Sabah, MALAYSIA

## Abstract

**Background:**

Blood donation is vital for health systems, yet global shortages persist due to low donor participation. Understanding the psychological determinants of donation is critical to improving recruitment and retention. The Theory of Planned Behavior has been widely applied to predict donation intention and behavior.

**Objective:**

This review aimed to map and synthesize the evidence on blood donation practices and behavioral intentions through the lens of the Theory of Planned Behavior, identifying key determinants, barriers, facilitators, and research gaps.

**Methods:**

We conducted a scoping review guided by PRISMA-ScR. Major databases (PubMed, Web of Science, CINAHL, Cochrane, Hinari, and the WHO library) and grey literature were searched for studies published between January 1, 2004, and August 31, 2025. Eligible studies applied the theory of planned behavior constructs to blood donation practices across different populations and settings.

**Results:**

Nineteen studies met the inclusion criteria. Most (n = 12) focused on students, while others examined healthcare workers or established donors. Cross-sectional designs predominated. Theory of planned behavior constructs, including attitude, perceived behavioral control, self-efficacy, moral norms, and anticipated regret were strong predictors of intention to donate blood. However, intention alone was insufficient to consistently predict actual donation behavior. Reported barriers included fear, low self-efficacy, and systemic challenges; facilitators included altruism, positive attitudes, social influence, and prior donation experience.

**Conclusion:**

Theory of planned behavior provides a robust framework for understanding blood donation intentions, but augmenting it with moral norms and past behavior may improve the prediction of actual behavior. Interventions should target attitudes, perceived behavioral control, self-efficacy, and supportive social norms, while addressing systemic barriers, to strengthen voluntary donor recruitment and retention.

## Introduction

Blood donation is a cornerstone of modern healthcare systems, ensuring the availability of safe blood and blood products for life-saving procedures, including surgery, trauma care, cancer treatment, and maternal health emergencies [1]. Despite the critical need, the global blood supply remains insufficient in many low- and middle-income countries, where demand often exceeds collection capacity, contributing to preventable morbidity and mortality [2]. Even in high-income settings, where transfusion services are better developed, voluntary donor recruitment and retention remain persistent challenges [3].

Understanding the psychological and behavioral mechanisms that influence blood donation is there fore essential for improving donor mobilization strategies. While many individuals express willingness to donate, this intention does not always translate into actual behavior, creating a significant “intention–behavior gap” [4]. Factors such as fear of needles, misconceptions about health consequences, lack of knowledge, and structural barriers (e.g., inconvenient donation sites) contribute to low participation rates [5,6]. Conversely, altruism, moral obligation, and prior donation experiences are frequently identified as facilitators [7].

The Theory of Planned Behavior (TPB), proposed by Ajzen (1991), has emerged as one of the most widely applied frameworks for explaining health-related behaviors, including blood donation. According to TPB, intention to perform a behavior is shaped by three core constructs: attitude (positive or negative evaluation of donation), subjective norms (perceived social pressure to donate), and perceived behavioral control (PBC) (confidence in one’s ability to donate). These constructs together predict intention, which in turn influences behavior. Extensions of TPB incorporating self-efficacy, moral norms, and anticipated regret have been shown to strengthen predictive power for blood donation practices [8–10].

Given the increasing number of studies applying TPB to blood donation across diverse populations, students, health professionals, community members, and established donors, there is a need to synthesize the evidence systematically. While prior systematic reviews have assessed predictors of donation behavior [11,12]. No comprehensive scoping review has mapped the extent, range, and nature of TPB applications to blood donation practices worldwide.

Therefore, this scoping review aims to summarize existing evidence on blood donation practices and behavioral intentions using the Theory of Planned Behavior. Specifically, it maps how TPB has been applied in different populations and contexts, identifies key determinants of donation intention and behavior, and highlights barriers, facilitators, and research gaps. Insights from this review can inform theory-driven interventions to enhance voluntary blood donor recruitment and retention strategies globally.

## Materials and methods

### Literature search and search methods

Our review aimed to identify the available evidence and provide an overview of the scoping review objectives on the current state of knowledge regarding blood donation practices, applying the Theory of Planned Behavior (TPB). This review was conducted following the PRISMA-ScR (Preferred Reporting Items for Systematic reviews and Meta-Analyses extension for Scoping Reviews) checklist, and it was guided by the Joanna Briggs Institute (JBI) scoping review guidance [13,14]. A search was conducted for published and unpublished (grey) literature in the following electronic databases: MEDLINE (PubMed), Cochrane Library, Web of Science, CINAHL, Hinari, and the World Health Organization (WHO) library. Moreover, grey literature was searched on Google, Google Scholar, and World Wide Science.org. All databases and grey literature sources were searched for studies published between January 1, 2004 and August 31, 2025**.**The final search across all databases was conducted on 31 August 2025.We used different combinations of keywords and text to build the search strategy and identify relevant articles (S1 File).

To identify potentially relevant literature, a hand search was also conducted of the reference lists of included studies and websites such as the WHO, International Federation of Red Cross and Red Crescent Societies (IFRC), and Global Database on Blood Safety. Additional grey literature was identified through targeted searches of dissertations, theses, and conference abstracts.

### Scoping review rsearch question

We used a population, concept, and context (PCC) framework developed by the JBI to determine the eligibility of our primary research question. The primary research question for this scoping review was:

“How has the Theory of Planned Behavior been applied to understand or predict blood donation practices among different populations?”

The secondary research question was:

“To what extent can insights from the Theory of Planned Behavior be incorporated by health professionals, blood bank managers, and policy makers in designing strategies to improve voluntary blood donation practices?”

This study used the PCC format to align the study selection with the aforementioned research questions (Table 1).

**Table 1:** Eligibility of studies according to the participant, concept, and context (PCC) framework.

Criteria	Elements
**P: participants**	All categories of individuals who are eligible for or have participated in blood donation (General public, university students, health professionals, community members, or voluntary donors).
**C: concept**	Studies that applied or assessed the Theory of Planned Behavior (TPB) in relation to blood donation practices, including attitudes, subjective norms, perceived behavioral control, and intention to donate blood. Interventions or programs designed using TPB constructs to promote blood donation were also included.
**C: context**	All countries and settings (low-, middle-, and high-income) where blood donation practice has been studied in relation to the Theory of Planned Behavior.

### Study selection criteria

#### Inclusion criteria.

- Studies focusing on blood donation practices among any population.
- Studies that explicitly applied or tested the Theory of Planned Behavior (TPB) or any of its constructs (attitude, subjective norm, perceived behavioral control, and intention) in relation to blood donation.
- Both qualitative, quantitative, and mixed-methods studies.
- Published and unpublished (grey literature) from January 1, 2004 to August 31, 2025.

#### Exclusion criteria.

- Publications not in English.
- Studies not applying the Theory of Planned Behavior or its constructs to blood donation.
- Studies for which full-text could not be obtained, even after contacting authors.

### Data extraction and management

Data were extracted using a standardized data extraction spreadsheet, which captured essential characteristics of each study. These included the author(s) and year of publication, the country or region where the study was conducted, the study population, and the study design and methodology. Additionally, the extraction form recorded the Theory of Planned Behavior (TPB) constructs assessed in the study, such as attitude, subjective norms, perceived behavioral control, intention, and behavior. Key findings were also documented, including predictors of blood donation, interventions tested, and reported outcomes. Data extraction was carried out independently by two reviewers (GL and ANY). Titles and abstracts were screened independently by two reviewers. Inter-rater agreement during the title and abstract screening phase was assessed using Cohen’s kappa statistic (κ = 0.82), indicating substantial agreement. Any disagreements were resolved by discussion, and if unresolved, a third reviewer was consulted.

In this review, we distinguished between Perceived Behavioral Control (PBC) and self-efficacy as defined in the original studies. PBC refers to an individual’s perception of their overall control or ease in performing the behavior, while self-efficacy specifically captures confidence in the ability to perform the behavior under challenging circumstances. In some studies, these constructs were used interchangeably or treated as overlapping; in such cases, we reported the construct according to the authors’ definitions and noted the overlap.

### Study selection and reliability

The initial database searches were performed by two authors experienced in scoping reviews. Titles, abstracts, and full texts were screened independently by two reviewers. Any disagreements regarding article inclusion were discussed until consensus was reached; unresolved disagreements were adjudicated by a third reviewer.

To ensure consistency, one reviewer was blinded to the other’s decisions during article selection and data extraction. Discrepancies were resolved through consensus, with arbitration from a third reviewer where necessary.

### Data analysis

The methodological framework for this scoping review followed the Joanna Briggs Institute (JBI) guidance. Findings were presented using a dual approach. First, a numerical and descriptive analysis provided a basic quantitative summary of the included studies, detailing distributions by country, study population, study design, and the TPB constructs assessed, which were presented using tables. Second, a thematic synthesis offered a narrative account structured around key themes derived from the studies. These anticipated themes included predictors of blood donation intention and practice, barriers and facilitators of blood donation within the TPB constructs, the effectiveness of TPB-based interventions in promoting blood donation, and gaps in evidence regarding the application of TPB to blood donation. A codebook with definitions was prepared to guide the thematic synthesis, and the final narrative was organized according to the themes that emerged from the literature (Multimedia Appendix I (S1 Appendix)).

### Ethical approval and consent to participate

Ethical approval and informed consent were not required for this study because it is a scoping review based exclusively on previously published literature

## Results

### Flow of the search and study characteristics

A total of 1,145 potentially relevant studies were identified from the initial database search (PubMed = 752, Google Scholar [advanced] = 42, Google = 88, CINAHL = 54, Hinari = 17, and Web of Science = 192). After removing duplicates, 328 studies remained for screening. Based on the review of titles (n = 210) and abstracts (n = 62), the majority were excluded as not being directly related to blood donation or TPB. Overall, 57 studies were eligible for full-text assessment.

**Fig 1:**
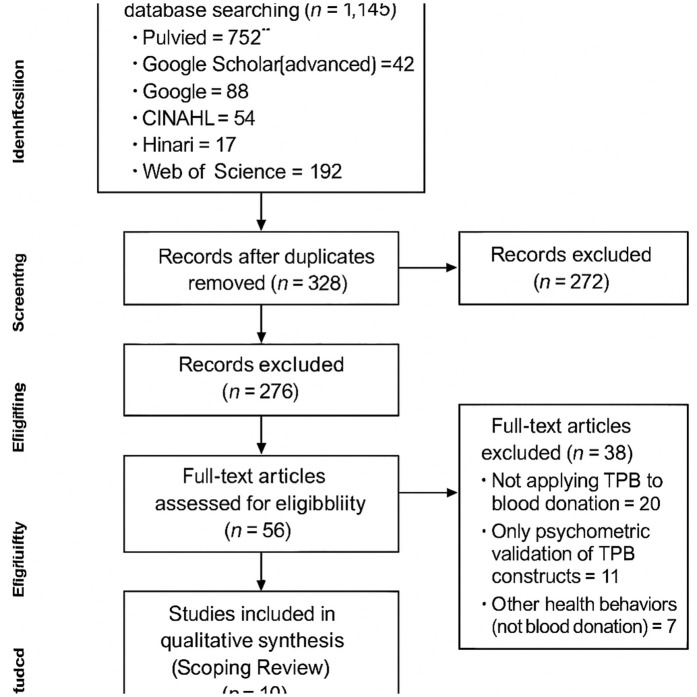
Flow diagram for the scoping review process adapted from the PRISMA (Preferred Reporting Items for Systematic reviews and Meta-Analyses) statement.

After reviewing the full texts, 38 studies were excluded for the following reasons: did not apply the Theory of Planned Behavior framework to blood donation (n = 20), focused only on psychometric validation of TPB constructs without assessing practice (n = 11), or addressed other health behaviors unrelated to blood donation (n = 7). Finally, 19 articles were included in this scoping review (Fig 1).

Of the studies included in this review, 7 addressed blood donation intention and behavior from the perspective of potential donors, including university and secondary school students, healthcare workers, and adults in the general population across China, Ethiopia, Uganda, Malaysia, and India [15–21]. These studies consistently showed that perceived behavioral control, self-efficacy, and attitude were key predictors of intention to donate blood.

Blood donation intention and behavior were also examined in 5 studies from the perspective of established or experienced blood donors, including voluntary whole blood and plasmapheresis donors in Australia, Iran, and China [22–26]. These studies highlighted that moral norms, anticipated regret, and past donation behavior influenced intention and actual donation practices. In one Australian study, augmented TPB models explained up to 70% of actual donation behavior among established donors [22].

The role of knowledge, awareness, and subjective norms in shaping intention to donate blood was addressed in 4 studies, including university students and healthcare workers in China, Malaysia, and India [18,19,24,26]. Findings indicated that greater awareness of the benefits of donation, such as saving lives, significantly increased intention to donate. At the same time, subjective norms played a variable role depending on the population studied.

Finally, 3 studies applied TPB to explore blood donation intention among health care providers, clinicians, and educators [19,26,27]. These studies demonstrated that perceived knowledge, hierarchical position, and professional experience influenced clinicians’ intentions to prescribe or participate in blood donation, underscoring the need to address both individual and systemic factors to enhance donation behavior (Multimedia Appendix II (S2 Appendix)).

### Characteristics of the included studies

In this review, 4 studies originated from Africa, namely Ethiopia and Uganda [17,20,21,28], 7 studies were from Asia, namely China, Pakistan, India, and Malaysia [15,16,18,19,27,29,30], 8 studies were from other continents, including Australia, New Zealand, the United Kingdom, and Iran [4,22,25,31–35]. A qualitative approach was used in 1 study [28], 1 study used a literature review [4], and the remaining 17 studies used a quantitative cross-sectional design (Table 2).

**Table 2:** Characteristics of the included studies.

Variable	Category	n (%)
Perspective	Potential blood donors (students/adults)	7 (37)
	Established/experienced donors	5 (26)
	Health care providers/clinicians	3(16)
	University students and health care providers	4 (21)
Publication type	Original research article	18 (94.7)
	Literature review	1 (5.3)
Study design	Cross-sectional	17 (89.5)
	Qualitative	1 (5.3)
	Literature review	1 (5.3)
Themes in the studies	Intention to donate blood	19 (100)
	Actual donation behavior	10 (52.6)
	Determinants of donation (attitude, self-efficacy, subjective norms, knowledge, moral norm)	19 (100)
	Donor retention	6 (31.6)

### Themes in the studies

Based on the review of the included articles, the primary focus of the studies broadly fit into 5 key themes, namely: (1) determinants of blood donation intention, (2) predictors of actual donation behavior, (3) barriers to blood donation, (4) facilitators of blood donation, and (5) perspectives of donors and health care providers.

### Determinants of blood donation intention

Determinants of intention to donate blood were the most commonly examined theme across studies. Attitudes toward donation, perceived behavioral control/self-efficacy, subjective norms, and moral norms were consistently reported as predictors of donation intention [15–19,22,29,30,33,34,36,37].Positive, high self-efficacy, and supportive social norms were associated with greater intention to donate, while anxiety and low confidence reduced intention. Knowledge about blood donation also played a key role in shaping intention, particularly among university students and healthcare workers [18,19,30,37].

### Predictors of actual donation behavior

Several studies examined how intention translated into actual donation behavior. Past donation history, anticipated regret, and donor identity were found to predict whether individuals acted on their intention [22,23,25,29,32,34]. However, some studies noted a gap between intention and behavior, highlighting that intention alone was insufficient to ensure actual blood donation [32,34].

### Barriers to blood donation

Barriers to donation were identified at individual, social, and systemic levels. Individual barriers included fear of needles, concerns about health effects, lack of knowledge, and low perceived control [17,29,30]. Social barriers included limited encouragement from peers or family and a lack of visible role models [17,36]. Systemic barriers included inconvenient donation sites, limited donation opportunities, high patient or donor flow, and insufficient communication from blood banks [28,32,34,37].

### Facilitators of blood donation

Facilitators were factors that enabled or motivated individuals to donate blood. Key facilitators included prior positive donation experience, supportive social norms, moral obligation, altruism, and awareness of the lifesaving impact of blood donation [15,18,19,22,29,31]. Educational interventions, outreach campaigns, and accessible donation centers were also reported as important facilitators for increasing intention and actual donation behavior [16,18,19,28,30,37].

### Perspectives of donors and health care providers

Studies examined the perspectives of potential donors, established donors, and healthcare providers. Potential donors, particularly students, often expressed high intention but low actual donation rates, indicating a gap between willingness and action [15–17,30,36]. Established donors highlighted the importance of moral norms, and social support in sustaining donation behavior [22,25,31]. Healthcare providers and clinicians contributed insights on knowledge, professional experience, and institutional factors that influence donor recruitment and blood transfusion practices [4,30,37].

## Discussion

### Principal findings

Unlike previous reviews, which primarily focused on single populations or limited geographical areas, this scoping review provides a comprehensive global mapping of studies applying the Theory of Planned Behavior to blood donation**.** By doing so, it fills a gap in understanding the intention–behavior relationship and cross-contextual determinants of blood donation.

The scoping review explored the available literature on blood donation behavior based on the perspectives of potential and actual blood donors, as well as health care providers involved in donor recruitment and management, in a global context. The included studies predominantly originated from countries with active blood donation campaigns, including Ethiopia, Nigeria, China, and Iran, reflecting the growing attention to voluntary blood donation as a public health priority [15–19,22,29,30,33,34,36,37]. Most of the studies (over 80%) were published within the last five years, suggesting increasing recognition of the role of behavioral theories, particularly the Theory of Planned Behavior (TPB), in understanding blood donation intention and practice [17,30,32,34,36].

Positive attitudes toward donation, high self-efficacy, and supportive social norms were consistently associated with greater intention to donate, while negative beliefs, fear, and low confidence reduced intention [17,29,30]. Knowledge about blood donation and prior donation experience also strengthened intention and actual donation behavior [18,22,31].

However, the predictive power of TPB constructs varies across populations and contexts. Among students and general populations, attitudes and perceived behavioral control were the strongest predictors of intention, whereas among established donors, moral norms and past donation experience more strongly influenced actual donation behavior. Regional differences also emerged, with Asian studies emphasizing knowledge and awareness, and African studies highlighting systemic barriers such as site accessibility and donor flow. These inconsistencies point to under-explored areas, including healthcare providers and low-resource settings, where further research could refine theory-driven interventions.

Blood donation behaviors have direct impacts on the adequacy of the blood supply and overall public health outcomes. Actual donation behavior was influenced not only by intention but also by logistical, social, and personal factors. For example, the availability of accessible donation sites, prior positive donation experiences, moral obligation, and altruism facilitated conversion from intention to action [18,19,22,28,31]. However, a gap between intention and practice remains a significant challenge, emphasizing the need for strategies that bridge this intention–behavior gap [32,34].

Barriers to blood donation identified in this review included fear of needles, concerns about health effects, low awareness, inconvenient donation sites, high donor flow, and lack of institutional support [28,29,32,34,37]. Social barriers, such as limited encouragement from family and peers, and systemic barriers, including insufficient outreach and donor recruitment programs, also hindered donation [17,28,36]. Conversely, facilitators included education campaigns, social support, reminders, and incentives, which increased motivation and actual donation behavior [16,18,19,30,37].

Perspectives from donors and health care providers highlighted the importance of empathy, communication, and trust-building. Potential donors, particularly students, reported high intention but low actual donation rates, indicating the need for interventions that enhance self-efficacy and reduce perceived barriers [15–17,30,36]. Established donors emphasized the influence of social support, moral norms, and donor identity in sustaining donation behavior [22,25,31]. Health care providers underscored the importance of institutional support, proper donor management, and continuous education to improve recruitment and retention [4,30,37].

Empirical findings consistently show that self-efficacy, attitudes, and social norms predict donation intention, while normative implications suggest that interventions should target donor confidence, positive attitudes, supportive social norms, and systemic barriers to enhance actual blood donation, particularly in low-resource settings.

Overall, the findings suggest that integrating TPB-informed interventions into blood donation campaigns could enhance intention, bridge the gap to actual donation behavior, and increase overall blood supply. These insights can inform the design of future interventions and donor recruitment strategies, although the review does not prescribe specific intervention approaches. Efforts should focus on strengthening knowledge, building positive attitudes, enhancing self-efficacy, engaging social norms, and addressing logistical barriers to optimize voluntary blood donation. Future research should explore under-studied contexts and populations, examine the translation of intention into actual donation behavior, and evaluate the effectiveness of theory-informed interventions.

### Strengths and limitations

This scoping review has several strengths. First, its broad approach provides an extensive overview of the existing literature, capturing a wider context than might be possible with a systematic review. Second, adherence to the PRISMA-ScR and Joanna Briggs Institute (JBI) guidelines enhanced the transparency and rigor of the review process.. While this approach allows comprehensive mapping of available evidence, it limits the ability to evaluate the strength, certainty, or risk of bias of the findings. Therefore, the results should be interpreted as a descriptive synthesis of the existing literature rather than as confirmatory evidence of causal relationships. Additionally, restricting the review to articles published in English may have excluded relevant studies published in other languages, potentially introducing language bias.

### Conclusion

Based on the evidence from included studies, interventions that enhance knowledge about blood donation are supported by findings from studies in China, Malaysia, and India, which show greater awareness increases donation intention. Programs aimed at strengthening self-efficacy are justified by multiple studies showing its consistent role in predicting intention and behavior. Social norms interventions may be considered, though their effect varies by population. Finally, addressing systemic barriers such as access to donation centers is suggested, based on qualitative evidence highlighting fears, misconceptions, and logistical constraints.

## Supporting information

S1 Appendix Multimedia Appendix I: Codebook for thematization of the study. (DOCX)

S2 Appendix Multimedia Appendix II: Study characteristics. (DOCX)

S1 File Full search strategies and search dates. (DOCX)

S2 File Preferred reporting items for the scoping review. (PDF)
