# Supplementary material for: Blood donation practices and behavioral intentions: A scoping review using the theory of planned behavior
Source: PLoS One. 2026 Mar 12;21(3):e0333426. doi: 10.1371/journal.pone.0333426 (PMC12981459; doi:10.1371/journal.pone.0333426)
Supplement: S1 Appendix — (DOCX) [file pone.0333426.s001.docx]

| **Variable** | **Definition** | **Coding Options / Examples** |
| --- | --- | --- |
| Author | First author of the study | Text (e.g., Liu et al.) |
| Year | Year of publication | Numeric (e.g., 2023) |
| Country | Country where the study was conducted | Text (e.g., China, Ethiopia) |
| Continent | Continent classification | Africa, Asia, Europe, Oceania, Other |
| Perspective | Side of intervention studied | Individual/Recipient, Individual/Provider, Population |
| Population | Group receiving or involved in service | Text (e.g., University students, healthcare workers) |
| Sample Size | Number of participants | Numeric (e.g., 1165) |
| Study Design | Methodology | Cross-sectional, Descriptive cross-sectional, SEM cross-sectional, Qualitative, Review |
| Aim/Purpose | Main objective of the study | Text (brief summary) |
| TPB Constructs Studied | Theory of Planned Behavior constructs measured | Attitude, Subjective Norms, Perceived Behavioral Control / Self-efficacy, Moral Norm, Anticipated Regret, Knowledge, Awareness, etc. |
| Outcome Type | What was measured | Intention to donate, Actual donation behavior, Both |
| Key Findings | Main results relevant to TPB | Text summary (brief) |
| Behavioral Component | Whether study included actual behavior follow-up | Yes / No |
| Data Source | How information was collected | Survey, Interview, Literature review, Mixed-method |
| Coding Notes | Any special notes on coding | Example: “Self-efficacy” coded if confidence in ability to donate mentioned |

**Multimedia Appendix I:** Code book for thematization of the study
