# Supplementary material for: Blood donation practices and behavioral intentions: A scoping review using the theory of planned behavior
Source: PLoS One. 2026 Mar 12;21(3):e0333426. doi: 10.1371/journal.pone.0333426 (PMC12981459; doi:10.1371/journal.pone.0333426)
Supplement: S2 Appendix — (DOCX) [file pone.0333426.s002.docx]

| **Multimedia Appendix II : Study characteristics** | | | | | | | | | |
| --- | --- | --- | --- | --- | --- | --- | --- | --- | --- |
|  | | | | | | | | | |
| **S.N** | **Author.** | **year** | **country** | **perspective** | **The population for whom the service is delivered** | **Sample size** | **design** | **Aim/purpose** | **Key finding** |
| 1 | Liu et al | 2023 | China | Individual (young adults / potential blood donors) | Chinese university  students | 1165 | Cross sectional | To examine the determinants of blood donation intention among Chinese young adults using a modified and extended Theory of Planned Behaviour | - Attitude, subjective norms, and self-efficacy were positively associated with blood donation intention. Attitude and self-efficacy mediated the effects of anxiety, altruism, and social norms on intention, while attitude also mediated the relationship between altruism and social norms on intention |
| 2 | Kassie et al | 2020 | Ethiopia | Individual / Recipient | Potential blood donors (adults aged 18–65). | 515 | Cross sectional | To identify the determinants of blood donation intention among adults aged 18–65 and examine how attitude, subjective norms, perceived behavioral control, and past behavior predict intention to donate blood | - Direct perceived behavioral control, subjective norms, attitude, and past blood donation behavior were significant predictors of intention to donate blood. - The model explained 49% of the variance in blood donation intention. - The mean intention to donate blood was 3.02 ± 1.13. |
| 3 | Faqah et al | 2015 | Pakistan | Individual / Recipient | Medical students (novice blood donors) | 391 | Cross sectional | To determine the factors influencing the intention to donate blood among medical students who have never donated before, using the Theory of Planned Behavior. | Perceived behavioural control, anticipated regret, and attitude were the most influential predictors of blood donation intention; donation anxiety had a negative correlation. The model showed strong predictive power (R² = 0.811, adjusted R² = 0.807) |
| 4 | L.Bagot et al | 2015 | Australia | Individual / Recipient | Voluntary whole blood donors (recent and distant donors) | 693 | Cross sectional | To identify predictors that influence voluntary whole blood donors to become plasmapheresis donors using an extended Theory of Planned Behaviour | - Moral norm and self-efficacy positively influenced plasmapheresis donation intention, while a superordinate donor identity negatively affected intention. The extended TPB model effectively identified factors facilitating conversion from whole blood to plasmapheresis donation. |
| 5 | Holdershaw et al | 2011 | New Zealand | Individual / Recipient | Potential blood donors participating in a mobile blood drive in New Zealand | 45 | Cross sectional | To evaluate whether the Theory of Planned Behavior (TPB) can predict actual blood donation behavior and whether extended versions of TPB improve prediction compared to the standard model. | TPB predicts intentions to donate blood well but is much less effective at predicting actual donation behavior. Extending TPB with moral obligation, past behavior, or belief-based variables did not improve predictive ability. The study highlights the need for alternative approaches to identify factors that influence actual donation behavior |
| 6 | Veldhuizen et al | 2011 | Netherland | Individual / Recipient | Whole blood donors across all stages of donation experience | 11,480 | Cross-sectional | To investigate whether the same TPB variables consistently predict intention to donate across the entire donor career and different donation categories | - Self-efficacy is the main predictor of donation intention across all donation stages; cognitive attitude and moral norm contribute marginally; sex differences exist—self-efficacy is stronger for women, subjective norm predicts intention only in men; TPB variables consistently predict intention regardless of lifetime donation number |
| 7 | Giles et al | 2004 | United Kingdom | Individual / Recipient | Undergraduate students (potential blood donors) | 100 | Cross sectional | To examine the role of self-efficacy in predicting blood donation intention within the Theory of Planned Behavior framework | - Self-efficacy was the strongest predictor of intention, explaining 73% of the variance; it contributed more to intention prediction than past behavior and self-identity, highlighting its central role in blood donation behavior. |
| 8 | Masser et al | 2009 | Australia | Individual / Recipient | Established blood donors | 263 donors (182 completed follow-up) | Cross sectional with follow-up | To examine predictors of intention and actual blood donation behavior among experienced donors using an augmented Theory of Planned Behavior | Attitude, self-efficacy, and anticipated regret directly and indirectly predicted donation behavior via intention; moral norm, donation anxiety, and donor identity predicted intention indirectly; the model explained 51% of the variance in attitudes, 86% in intentions, and 70% in behavior; augmented TPB effectively identifies determinants of intentions and behavior among established donors |
| 9 | Akulume et al | 2024 | Uganda | Individual / Recipient | Secondary school students (potential blood donors) | 336 students | Cross-sectional | To apply the Theory of Planned Behavior to predict intention to donate blood among secondary school students | - About 75% of students intended to donate blood; perceived behavioral control was the strongest predictor (AOR = 6.35); subjective norms and attitudes were not significant; TPB constructs explained 15.5% of intention; external factors such as school location and knowing a donor also influenced intention |
| 10 | Parash et al | 2020 | Malaysia | Individual / Recipient | University students (potential blood donors) | 500 students | Cross sectional | To examine students’ behavioral intention to make voluntary blood donations using the Theory of Planned Behavior | - Knowledge was the strongest predictor of intention; all TPB constructs (attitude, subjective norm, perceived behavioral control) significantly influenced intention; awareness that donation saves lives increased students’ inclination to donate |
| 11 | Liao et al | 2023 | China | Individual / Provider | Clinicians (physicians) managing blood transfusions | 129 clinicians | Cross sectional | To develop and validate a TPB-based questionnaire to assess clinicians’ intentions to prescribe blood transfusions and identify influencing factors | - Perceived knowledge (β = 0.32) and subjective norms (β = 0.22) significantly influenced clinicians’ intentions; education level, hierarchy, and specialty affected perceived behavioral control; TPB constructs effectively explained determinants of clinicians’ blood transfusion intentions |
| 12 | Lim et al | 2020 | China | Individual / Recipient | Healthcare workers (potential blood donors) | 400 HCWs | cross-sectional | To identify factors influencing healthcare workers’ intention to donate blood using the Theory of Planned Behavior | - Perceived behavioral control and age significantly influenced intention; 60.3% of respondents had donated previously; SEM model explained 43% of variance in intention; findings highlight barriers and motivational factors for blood donation among HCWs |
| 13 | Negash et al | 2008 | Ethiopia | Individual / Recipient | Secondary school students (potential voluntary non-remunerated blood donors) | 450 students | cross-sectional | To determine prevalence and factors associated with voluntary blood donation among secondary school students using the Theory of Planned Behavior | - 70% of students were willing to donate voluntarily; actual past and regular donation rates were 10% and 1%, respectively; self-efficacy, attitude, and personal moral norm significantly predicted willingness; gender was associated with willingness (OR = 2.68) |
| 14 | Mansour et al | 2022 | Tehran | Individual / Recipient | Blood donors attending four blood transfusion centers | 316 participants | Crossectional | To predict behavioral intention to donate blood among donors in Tehran using the Theory of Planned Behavior and moral norms | - Planned behavior (r = 0.51) and moral norm (r = 0.15) were significantly correlated with intention; SEM showed planned behavior was a highly significant predictor (p < 0.001) and moral norm also significant (p < 0.05); attitude was not a significant predictor in this groupg |
| 15 | Saha et al | 2018 | India | Individual / Recipient | Potential voluntary blood donors in India | 45(1)0 respondents | Cross sectional | To examine and validate an integrative TPB framework incorporating voluntary function inventory (VFI) to predict intention toward voluntary blood donation | - TPB constructs—attitude, subjective norms, and perceived behavioral control—along with VFI components (value, social, career, enhancement) significantly predicted donation intention; the model provides a robust understanding of drivers of voluntary blood donation in India |
| 16 | Ferguson et al | 2007 | United kingdom | Individual / Recipient | Blood donors (general population) | Not applicable (literature review) | Review study | To examine theoretical developments in blood donor recruitment and retention, identify commonalities, and propose an integrated intervention approach | Emotional regulation (anticipated anxiety, vasovagal reactions) is key to donor motivation; intentions predict behavior, but enactment-focused interventions are underused; implementation intentions (“if-then” plans) are proposed to integrate social and behavioral science findings to improve donor recruitment and retention |
| 17 | Hasanzadeh et al | 2013 | Iran | Individual / Recipient | Blood donors at centers across Isfahan province | 600 | Cross-sectional | To determine factors influencing regular blood donation using the Theory of Planned Behaviour | - ‘Only 12.8% of donors returned within 6 months; regular donation was most strongly correlated with intention and self-efficacy (r = 0.577), followed by self-identity, subjective norms, and attitude; self-efficacy is a key factor for promoting donor retention |
| 18 | Ma et al | 2024 | China | Individual / Recipient | College and university students (potential blood donors) | 5,168 students. | Cross-sectional | To investigate determinants of blood donation among college and university students and identify the main influencing factors using SEM | - Overall donation rate was 24.71%; sociodemographic characteristics, health status, knowledge, and attitude positively influenced donation, with attitude being the primary factor; indirect effects of sociodemographic characteristics, health status, and knowledge were mediated by attitude; the model explained 22.22% of the variance in donation behavior; recommendations include enhancing knowledge and fostering positive attitudes for recruitment |
| 19 | Geleta et al | 2019 | Ethiopia | Population | Hawassa city residents | 12 FGD and 20 IDI | Qualitative (FGD & in-depth interviews) | To explore intention to donate blood among the Hawassa city population using the Theory of Planned Behaviour | Most participants were unaware of the minimum/maximum age for donation; main info sources were TV and friends; blood donation was seen as a humanitarian act; subjective norms (spouses) had little influence; the majority reported fears of health problems, anemia, and weakness. |
