# Supplementary material for: Blood donation practices and behavioral intentions: A scoping review using the theory of planned behavior
Source: PLoS One. 2026 Mar 12;21(3):e0333426. doi: 10.1371/journal.pone.0333426 (PMC12981459; doi:10.1371/journal.pone.0333426)
Supplement: S1 File — (DOCX) [file pone.0333426.s003.docx]

**S1 File. Full Search Strategies and Search Dates**

**Databases searched:** PubMed (MEDLINE), Web of Science, CINAHL, Cochrane Library
**Grey literature sources:** Google Scholar, Google (advanced search), WorldWideScience.org, WHO Library
**Language restriction:** English
**Time period:** January 1, 2004 – August 31, 2025
**Last search date:** 31 August 2025

**PubMed (MEDLINE)**

(("blood donation"[MeSH Terms] OR "blood donor*" OR "voluntary blood donation")

AND

("theory of planned behavior" OR "theory of planned behaviour" OR TPB

OR "behavioral intention" OR "behavioural intention"

OR attitude OR "subjective norm*" OR "perceived behavioral control" OR "self-efficacy"))

**Filters applied:**
• Language: English
• Publication dates: 2004/01/01 – 2025/08/31

**Web of Science**

TS = (("blood donation" OR "blood donor*" OR "voluntary blood donation")

AND

("theory of planned behavior" OR "theory of planned behaviour" OR TPB

OR "behavioral intention" OR "behavioural intention"

OR attitude OR "subjective norm*" OR "perceived behavioral control" OR "self-efficacy"))

**Filters applied:**
• Language: English
• Timespan: 2004–2025

**CINAHL**

(("blood donation" OR "blood donor*" OR "voluntary blood donation")

AND

("theory of planned behavior" OR "theory of planned behaviour" OR TPB

OR "behavioral intention" OR "behavioural intention"

OR attitude OR "subjective norm*" OR "perceived behavioral control" OR "self-efficacy"))

**Filters applied:**
• Language: English
• Published: 2004–2025

**Cochrane Library**

("blood donation" OR "blood donor*")

AND

("theory of planned behavior" OR TPB OR "behavioral intention")

**Filters applied:**
• Publication date: 2004–2025

**Grey literature**

Targeted searches were conducted using simplified keyword combinations such as:

“blood donation” AND “theory of planned behavior”
“blood donor” AND “behavioral intention”

Sources included dissertations, theses, conference abstracts, and reports from WHO and IFRC.
